# MCC950 suppresses NLRP3-dependent neuroinflammation and ameliorates cognitive decline in a rat model of cerebral small vessel disease

**DOI:** 10.4103/NRR.NRR-D-24-01055

**Published:** 2025-06-19

**Authors:** Meiyan Zhang, Xiaoyan Lan, Yue Gao, Shen Li, Guanda Qiao, Yajie Liang, Miroslaw Janowski, Piotr Walczak, Chengyan Chu

**Affiliations:** 1 Department of Neurology, Central Hospital of Dalian University of Technology, Dalian, Liaoning Province, China; 2 Department of Neurology and Psychiatry, Beijing Shijitan Hospital, Capital Medical University, Beijing, China; 3 Department of Diagnostic Radiology and Nuclear Medicine, University of Maryland School of Medicine, Baltimore, MD, USA

**Keywords:** astrocyte, autophagy, blood–brain barrier, cerebral small vessel disease, cognitive function, endothelial cells, microglia, neuroinflammation, NLRP3 inflammasome, white matter

## Abstract

Cerebral small vessel disease is a major vascular contributor to cognitive impairment and dementia. However, there remains a lack of effective preventative or therapeutic regimens for cerebral small vessel disease. In this study, we investigated the potential therapeutic effects of MCC950, a selective NOD-like receptor family pyrin domain-containing protein 3 inhibitor, on cerebral small vessel disease pathogenesis and cognitive decline in spontaneously hypertensive rats. Our results showed that chronic administration of MCC950 (10 mg/kg) to spontaneously hypertensive rats inhibited NOD-like receptor family pyrin domain-containing protein 3 inflammasome activation, thereby considerably suppressing the production of pyroptosis executive protein gasdermin D and pro-inflammatory factors, including interleukin-1β and -18. A decrease in astrocytic and microglial activation was also observed. We also found that MCC950 significantly inhibited autophagy. More importantly, behavioral assessment indicated that MCC950 administration ameliorated impaired neurocognitive function, which was associated with improvements in neuropathological hallmarks in the cerebral small vessel disease brain, such as blood‒brain barrier breakdown, white matter damage, and endothelial dysfunction. Thus, our findings revealed that the NOD-like receptor family pyrin domain-containing protein 3 inflammasome is a key contributor to the onset or progression of cerebral small vessel disease and suggested the potential of NOD-like receptor family pyrin domain-containing protein 3-based therapy as a potential novel strategy for treating cerebral small vessel disease.

## Graphical abstract

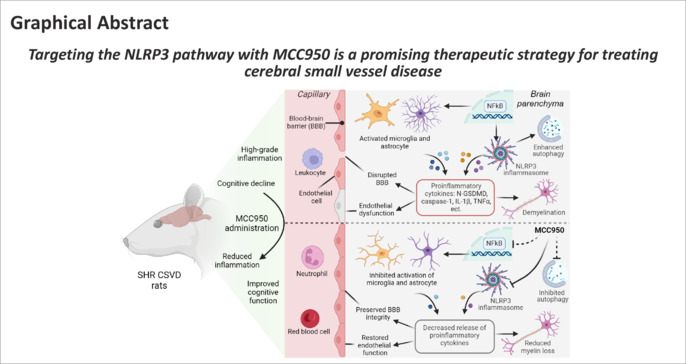

## Introduction

Cerebral small vessel disease (CSVD) refers to a disorder affecting perforating cerebral arterioles, capillaries, and venules of the brain (Wardlaw et al., 2013a). Neuroimaging features of CSVD on magnetic resonance imaging (MRI) include white matter hyperintensities (WMHs), lacunes, microbleeds, enlarged perivascular spaces, and subcortical infarcts (Wardlaw et al., 2013b). CSVD presents a spectrum of clinical manifestations, encompassing stroke, cognitive decline, mood irregularities, and abnormal gait. CSVD has emerged as a significant risk factor for stroke and is responsible for approximately a quarter of all ischemic strokes and the majority of hemorrhagic strokes (Kapasi et al., 2017; Wardlaw et al., 2019). Notably, it has surpassed other vascular issues to become the primary contributor to cognitive decline, with a staggering 50% involvement in dementia cases (Wardlaw et al., 2019). This places an immense burden on societies and healthcare systems, especially considering the increasing longevity and aging demographics (Gorelick et al., 2011). Unfortunately, effective preventative or therapeutic strategies for CSVD are lacking. Current approaches primarily revolve around lifestyle management, including smoking cessation and medical interventions such as antiplatelet and anti-hypertensive treatments, albeit with limited efficacy.

Endothelial dysfunction, blood‒brain barrier (BBB) breakdown and white matter degeneration are well-known pathological characteristics of the progression of CSVD (Gao et al., 2022). Neuroinflammation is increasingly recognized as another pivotal etiological factor for CSVD. A wide range of studies have shown that peripheral immune effects, including NK cell migration and glial (microglia and astrocyte) activation, within the CSVD-affected brain are closely associated with endothelial dysfunction and impaired integrity of the BBB and white matter (Jalal et al., 2012, 2015; Kaiser et al., 2014; Low et al., 2019). An *in vivo* rodent study revealed extensive white matter abnormalities and behavioral decline, in which the anti-inflammatory agent minocycline reversed white matter injury and improved cognitive performance (Jalal et al., 2015). More recently, a clinical investigation suggested that interleukin (IL)-1β and tumor necrosis factor (TNF) inhibitors could mitigate the severity of CSVD, highlighting the promise of neuroinflammation modulation as a new therapeutic strategy (Lv, 2024).

The NOD-like receptor (NLR) family pyrin domain-containing protein 3 (NLRP3) inflammasome has emerged as a key mediator of pathological inflammation via caspase 1-dependent release of the pro-inflammatory cytokines IL-1β and IL-18 along with gasdermin D (GSDMD)-mediated pyroptotic cell death (Swanson et al., 2019). The activation of caspase-1, in turn, facilitates the maturation and release of pro-inflammatory cytokines, including IL-1β and IL-18, and triggers inflammatory cell death, a phenomenon known as pyroptosis, which is mediated by N-GSDMD. Compelling evidence has demonstrated the contribution of the NLRP3 inflammasome to a variety of central nervous system (CNS) diseases associated with neuroinflammation, including stroke and Alzheimer’s disease (Heneka et al., 2018; Zhang et al., 2020). The NLRP3-mediated inflammatory response can result in white matter damage by impeding the differentiation of oligodendrocyte precursor cells and promoting the death of mature oligodendrocytes (Shao et al., 2021; Xiao et al., 2022; Cao et al., 2023; Renz et al., 2024). Pro-inflammatory cytokines such as IL-1β directly influence BBB permeability (Fetsko et al., 2024; Wang et al., 2014). In addition, a more recent report revealed that GSDMD activation was a key contributor to BBB breakdown (Wei et al., 2024a). Therefore, targeting NLRP3 might be a promising therapeutic strategy for treating CSVD, which remains unexplored.

Autophagy is a cytoplasmic degradative process that eliminates damaged or senescent cytoplasmic components, including dysfunctional organelles and misfolded proteins (Bonam et al., 2024; Zhang et al., 2025). Under steady-state conditions, autophagy is crucial for maintaining cellular homeostasis, development and survival. In response to stressors, autophagic activity may increase substantially. A growing body of evidence suggests that there is crosstalk between autophagy and inflammation (Cadwell, 2016). For example, studies on traumatic brain injury have shown that the induction of autophagy improves functional outcomes through its ability to mitigate extensive neuroinflammation (Hegdekar et al., 2023). The mechanisms involve the suppression of the NLRP3 inflammasome by limiting the availability of inflammasome activators (e.g., reactive oxygen species) and incorporating the inflammasome subunit (e.g., apoptosis-associated speck-like protein containing a caspase recruitment domain) into an autophagosome for degradation (Shi et al., 2012; Lu et al., 2022). Conversely, the NLRP3 inflammasome can bidirectionally modulate autophagy, with some evidence suggesting that activation of the inflammasome can enhance autophagy as a host defense mechanism (Tung et al., 2012; Bonam et al., 2024; Naeem et al., 2024). However, other evidence has indicated that the NLRP3 inflammasome and its resulting products (e.g., caspase 1) impede the autophagy process (Biasizzo and Kopitar-Jerala, 2020; Bonam et al., 2024). Therefore, investigating the interplay between NLRP3 and autophagy activity in the context of CSVD is highly important.

Hypertension is a well-known epidemiologic risk factor for CSVD. It results in pathological changes in cerebral small vessels, such as the loss of smooth muscle cells, deposits of fibrohyaline material, narrowing of the lumen, and thickening of the vessel wall (Gao et al., 2022). Vascular remodeling or injury eventually leads to impaired cerebral blood flow regulation, resulting in local hypoxia and ischemia in the brain parenchyma (Evans et al., 2021). These histopathological changes are characteristic of CSVD development and are also associated with endothelial dysfunction and increased BBB permeability (Mustapha et al., 2019). Hence, interest in developing hypertension-based CSVD models is increasing. A spontaneously hypertensive rat (SHR) strain was developed by inducing hypertension through brother-sister breeding of Wistar-Kyoto (WKY) rats, selectively mating male rats with significant hypertension and female rats with mild hypertension (Okamoto and Aoki, 1963). The SHR has been increasingly used as an animal model of CSVD because of its similar behavioral changes and neuropathological characteristics to those of human CSVD, including BBB disruption and white matter damage (Jalal et al., 2012; Kaiser et al., 2014; Wei et al., 2024b).

In the present study, SHR rats were used as a model of early-onset CSVD, and WKYs were used as the normotensive control group. We aimed to characterize the NLRP3-mediated inflammatory response in model animals. We hypothesize that MCC950, a specific and potent NLRP3 inhibitor, mitigates neuropathological changes, including a compromised BBB, white matter degeneration and endothelial dysfunction, ultimately restoring cognitive performance in CSVD. This study lays the groundwork for validating NLRP3-based therapy as a novel treatment approach for CSVD.

## Methods

### Animals and treatment

**Figure 1:**
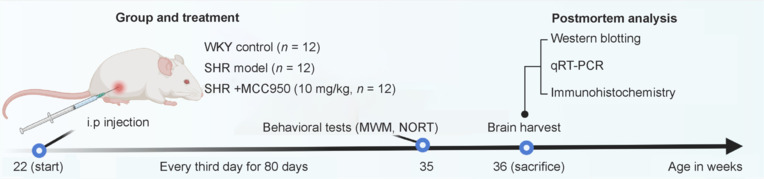
Experimental schematic diagram. i.p: Intraperitoneal; MWM: Morris water maze; NORT: novel object recognition test; qRT-PCR: quantitative reverse transcription-polymerase chain reaction; SHRs: spontaneously hypertensive rats; WKYs: Wistar–Keuls rats.

All experiments were performed in accordance with the protocol approved by the Institutional Animal Care and Use Committee at Central Hospital of Dalian University of Technology (approval No. TN2024-186-01; approval date: January 25, 2024). Specific-pathogen-free male SHR rats (*n* = 24) and WKY rats used as control strains (*n* = 12, both 8–10 weeks old, 150–200 g, Charles River, Beijing, China) were used in this study. Only male rats were used because SHR males consistently have higher blood pressure than females, resulting in a more severe phenotype (Reckelhoff et al., 1999). The rats were housed two per cage in a room maintained at a temperature of 21 ± 1°C and humidity of 50% ± 20% on a 12-hour light/dark cycle, with food and water provided ad libitum. All the rats were treatment naïve. SHRs were randomly divided into two experimental groups: the SHR model group (*n* = 12) and the MCC950-treated SHR group (*n* = 12). WKY rats (*n* = 12) were used as the WKY control group. SHR rats were intraperitoneally injected with MCC950 (a selective NLRP3 inhibitor; MedChemExpress, Monmouth Junction, NJ, USA, 10 mg/kg body weight in PBS) starting at 22 weeks of age, and the administration was carried out every third day for 80 days as described previously (He et al., 2020). At 36 weeks of age, following the behavioral tests, rats (*n* = 4) were randomly selected from each group and sacrificed through intranasal administration of 5% isoflurane (Shenzhen Reward Life Science Co., Ltd., Shenzhen, China). The brains were harvested for immunohistochemical and biological molecular studies. The experimental design and timeline are schematically represented in **Figure 1**.

### Behavioral tests

Behavioral assessments were carried out at 35 weeks. The Morris water maze (MWM) was used to evaluate spatial learning and memory as described previously (Wang et al., 2023). Briefly, the test in our study comprised the following two components: a consecutive place navigation test and a spatial probe test. For the place navigation test, the rats were placed in the pool to familiarize themselves with the environment and underwent a five-day training regimen. The escape latency, which refers to the time it took the animals to reach the platform and stay there for more than 15 seconds, was measured during training. The spatial probe test was performed 24 hours after the last training session (day 5). The platform was removed, and the animals were gently placed in the quadrant opposite the target quadrant. They were allowed to swim freely for 30 seconds. Water maze tracking software was used to record the duration spent in the target quadrant and track their search patterns for the platform. The novel object recognition test (NORT) was carried out according to a previously described protocol to evaluate non-spatial recognition memory (Kaiser et al., 2014). Briefly, the test procedure includes three sessions: habituation, training, and retention. The animals were allowed to habituate to the open field arena for 10 minutes on the first day. On the second day, they were placed in the arena to familiarize themselves with two identical objects for 2 consecutive days. Four hours after the second habituation training, the arena was equipped with the familiar and novel objects, and the animals were allowed to explore the area for 5 minutes. The discrimination ratio (interaction time with novel object interaction/total object interaction) was calculated, and a higher ratio was considered to reflect better performance.

### Immunohistochemical staining

The rats were anesthetized via inhalation of excessive isoflurane as described above. Transcardial perfusion was then performed using 5% sucrose in PBS, followed by 4% paraformaldehyde (PFA). The brains were subsequently dissected and fixed in 4% PFA overnight at 4°C and then cryopreserved in 30% sucrose. The brains were sliced into 30-μm thick coronal sections using a cryostat (RWD Life Science, Shenzhen, China). For immunofluorescence staining, the sections were blocked with 5% bovine serum albumin (BSA) in 0.1% Triton X-100 for 1 hour at room temperature. The sections were then incubated with primary antibodies overnight at 4°C. The antibodies used were as follows: ionized calcium binding adaptor molecule 1 (IBA1) (a marker for microglia), glial fibrillary acidic protein (GFAP) (a marker for astrocytes) and albumin for extraverted albumin in the brain. After being washed with PBS, the sections were incubated with Alexa Fluor® 594-conjugated goat anti-rabbit IgG (H&L) or Alexa Fluor® 488-conjugated goat anti-rabbit IgG (H&L) secondary antibodies for 2 hours at room temperature. Aqueous nonfluorescing mounting medium was added to the sections (Immu-Mount, 9990402; Thermo Scientific, Kalamazoo, MI, USA) for microscopic observation. The specific antibodies used are listed in **Table 1**. To visualize the myelin sheath and analyze white matter density in the corpus callosum, eriochrome cyanine staining was performed as described previously (Chu et al., 2020). Briefly, the slides were dried thoroughly in an oven for 2 hours at 50°C. The slides were dehydrated in 95% and 70% ethanol followed by washing with distilled water. Then, the sections were stained for 15 minutes with an eriochrome cyanine solution containing 0.2% eriochrome cyanine R (3275, Sigma, St. Louis, MO, USA), 0.4% FeCl_3_, and 0.5% H_2_SO_4_. Section development was performed by alternating exposure to 0.1% NH_4_OH for 5–10 seconds and rinsing in distilled water for 30 seconds until white matter (deep blue) was clearly differentiated from gray matter. After differentiation, the slides were placed in xylene for 10 minutes and mounted with coverslips using Krystalon. Immunofluorescent and histochemical images were captured using an inverted Olympus microscope (Olympus Corporation, Shinjuku, Japan). For the quantification of GFAP- and IBA1-positive cells, the myelin index (mean gray value) was calculated based on Eriochrome cyanine staining using ImageJ Version 1.54 (NIH, Bethesda, MD, USA).

**Table 1:** Antibodies used in the study

Antibody	Host	Dilution	Application	Supplier	Cat#	RRID
**Primary antibodies**						
IBA1	Rabbit	1:250	IF	Wako, Osaka, Japan	019-19741	AB_839504
GFAP	Rabbit	1:250	IF	Dako, Glostrup, Denmark	Z0334	AB_10013382
Albumin	Rabbit	1:100	IF	Novus, Centennial, CO, USA	NBP1-32458	AB_10003946
Claudin-5	Rabbit	1:500	WB	Bioworld, Nanjing, China	BS1069	AB_1664057
ZO-1	Rabbit	1:1000	WB	Invitrogen, Carlsbad, CA, USA	61-7300	AB_2533938
eNOS	Rabbit	1:1000	WB	Abcam, Cambridge, MA, USA	ab199956	AB_3094576
GSDMD	Rabbit	1:1000	WB	Abcam	ab219800	AB_2888940
NF-κB p65	Rabbit	1:1000	WB	Abcam	ab32536	AB_776751
phospho-Ser536-NF-κB	Rabbit	1:1000	WB	Abcam	ab76302	AB_1524028
MBP	Rabbit	1:1000	WB	Abcam	ab218011	AB_2895537
NLRP3	Rabbit	1:1000	WB	Abcam	ab263899	AB_2889890
p-eNOS	Rabbit	1:500	WB	Affinity, Changzhou, Jiangsu, China	AF3247	AB_2834673
Cleaved-caspase1	Rabbit	1:500	WB	Affinity	AF4005	AB_2845463
IL-1β	Rabbit	1:500	WB	Affinity	AF5103	AB_2837589
IL-6	Rabbit	1:500	WB	Affinity	DF6087	AB_2838055
Il-8	Rabbit	1:500	WB	Affinity	DF6252	AB_2838218
TNF-α	Rabbit	1:500	WB	Affinity	AF7014	AB_2835319
LC3A/B	Rabbit	1:500	WB	Affinity	AF5402	AB_2837886
SQSTM1/p62	Rabbit	1:500	WB	Affinity	AF7875	AB_2844239
**Secondary antibodies**						
Alexa-594	Goat	1:250	IF	Invitrogen	A-11012	AB_2534079
Alexa-488	Goat	1:250	IF	Invitrogen	A-11034	AB_2576217
Rabbit IgG	Goat	1:3000	WB	Abcam	Ab6721	AB_955447

eNOS: Endothelial nitric oxide synthase; GFAP: glial fibrillary acidic protein; IBA1: ionized calcium binding adaptor molecule 1; IF: immunofluorescence; MBP: myelin basic protein; NF-κB: nuclear factor kappa B; p-eNOS: phospho-eNOS; WB: western blot; ZO-1: zonula occludens-1.

### Western blotting

The rats were sacrificed with an overdose of isoflurane as described above and then euthanized by cervical dislocation. The whole brains were dissected immediately and incubated in lysis buffer containing phenylmethylsulfonyl fluoride (PMSF) and protease inhibitors for 30 minutes, followed by centrifugation at 1690 × *g* at 4°C for 15 minutes. The supernatant was collected to measure the protein concentration using a bicinchoninic acid (BCA) protein assay kit (Thermo Fisher Scientific). Equal amounts of protein were loaded in a 10% SDS‒PAGE gel (Bio-Rad Laboratories, Hercules, CA, USA). Following electrophoresis, the proteins in the gel were transferred onto PVDF membranes (Millipore, Billerica, MA, USA), which were subsequently blocked for 2 hours with 5% skimmed milk (w/v) in Tris-buffered saline (TBST) at room temperature. The membranes were incubated with the following primary antibodies at 4°C overnight: Claudin-5, Zonula occludens-1 (ZO-1), endothelial nitric oxide synthase (eNOS), phospho-eNOS (p-eNOS), GSDMD, nuclear factor kappa B (NF-κB) p65, phospho-Ser536-NF-κB p65, NLRP3, cleaved-caspase-1, IL-1β, IL-6, IL-18, TNF-α, myelin basic protein (sMBP), SQSTM1/p62, and LC3A/B. The membranes were rinsed with TBST three times and incubated with the corresponding secondary antibodies for 2 hours at room temperature. The immunoblots were then incubated with enhanced chemiluminescence (ECL) reagents (Pierce, Rockford, IL, USA) for imaging, and the band signals were quantified using ImageJ. The specific antibodies used are listed in **Table 1**.

### Quantitative reverse transcription‒polymerase chain reaction

The rats were sacrificed with an overdose of isoflurane, and the brains were harvested immediately. TRIzol (Invitrogen, Carlsbad, CA, USA) was used to extract total RNA from brain samples, which was reverse transcribed into cDNA via the RevertAid First Strand cDNA Synthesis Kit (Thermo Fisher Scientific). Quantitative reverse transcription‒polymerase chain reaction (qRT-PCR) was performed using the StepOnePlus^TM^ Real-Time PCR System (Thermo Fisher Scientific, Waltham, MA, USA). The reaction conditions were as follows: initial denaturation at 95°C for 5 minutes, followed by 45 cycles of denaturation at 95°C for 15 seconds and annealing/extension at 60°C for 35 seconds. The primer sequences are listed in **Table 2**. This experiment was performed in triplicate, and the mRNA expression was determined using the 2^–ΔΔCt^ method and normalized to that of GAPDH as a control.

**Table 2:** Sequences of primers used for quantitative reverse transcription‒polymerase chain reaction

Gene	Forward (5&–3&)	Reverse (5&–3&)	Size (bp)
*IL-18*	TGC CAT ACC AGA AGA AGG CTC	AGT GAA GTC TGC CAA AGT GGT	122
*IL-1β*	AGG ACC CAA GCA CCT TCT TT	GTC GTC ATC ATC CCA CGA GT	74
*TNF-α*	GAG GCG CTC CCC AAA AAG AT	GCC ACG AGC AGG AAT GAG AA	83
*IL-6*	CCA GTT GCC TTC TTG GGA CT	CTG GTC TGT TGT GGG TGG TA	103
*GAPDH*	AGG TTG TCT CCT GTG ACT TCA A	CTG TTG CTG TAG CCA TAT TCA TTG	130

GAPDH: Glyceraldehyde-3-phosphate dehydrogenase; IL: interleukin; TNF-α: tumor necrosis factor-alpha.

### Statistical analysis

No statistical methods were used to predetermine sample sizes; however, our sample sizes are similar to those reported in previous publications (Lesniak et al., 2019a, b; Zhang et al., 2024). The data are presented as the mean ± SD. The normality of the data was determined with the Shapiro‒Wilk test. For normally distributed data, one-way analysis of variance with Tukey’s *post hoc* test was used for multiple groups, and an unpaired *t-*test was used when two groups were compared. The latency from the MWM test were analyzed via repeated-measures analysis of variance, with escape latency on 5 consecutive days as the repeated-measures variable and “group” as the independent variable. Gene expression data were log2 transformed before being plotted in GraphPad Prism 8.0 (GraphPad Software, San Diego, CA, USA, www.graphpad.com) and displayed as heatmaps. All analyses were performed using GraphPad Prism 8.0, and a *P* value of less than 0.05 was considered statistically significant.

## Results

### NLRP3-mediated inflammatory pathway is activated in the brains of spontaneously hypertensive rats

**Figure 2:**
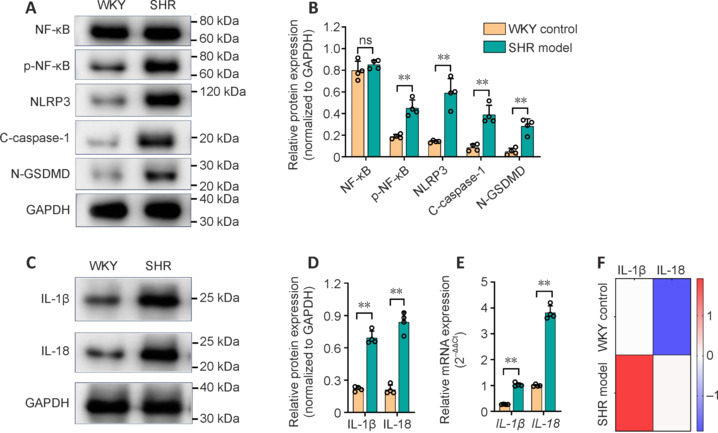
Quantification of NLRP3 inflammasome elements. (A, B) Representative immunoblots (A) and quantification (B) of the brain expression of NF-κB, p-NF-κB, NLRP3, C-caspase-1, and N-GSDMD in SHR model rats and WKY control rats. (C, D) Representative immunoblots (C) and quantification (D) of IL-1β and IL-18 in the brains of SHRs and WKY rats. (E) qRT-PCR analysis revealed greater gene expression of *IL-1*β and *IL-18* in SHRs than in WKY rats. (F) qRT-PCR-based heatmap of IL-1β and IL-18 gene expression in the brains of SHRs and WKY rats. The data are shown as the mean ± SD and were compared by an unpaired *t*-test, **P < 0.01, *n* = 4/group. All the experiments were repeated three times. C-caspase-1: Cleaved caspase-1; GAPDH: glyceraldehyde-3-phosphate dehydrogenase; IL-18: interleukin-18; IL-1β: interleukin-1 beta; NF-κB: nuclear factor kappa B; ns: not significant; p-NF-κB, phosphorylated NF-κB; N-GSDMD: N-terminal gasdermin D; NLRP3: NOD-like receptor family, pyrin domain-containing protein 3; qRT-PCR: quantitative reverse transcription‒polymerase chain reaction; SHR: Spontaneously hypertensive rat; WKY: Wistar‒Kyoto rat.

To determine the status of the NLRP3-mediated inflammatory pathway in the brain, we first performed western blotting to measure the protein levels of inflammasome elements (NLRP3, cleaved caspase-1, GSDMD, IL-1β, and IL-18) and NF-κB. A significant increase in the expression of NLRP3 was observed in the brains of the SHR model group compared with those of the WKY control group (*P* < 0.01; **Figure 2A** and **B**). Additionally, the expression of phosphorylated NF-κB (p-NF-κB) upstream and cleaved caspase-1 and N-terminal GSDMD (N-GSDMD) downstream was significantly greater than that in the WKY control group (*P* < 0.01; **Figure 2A** and **B**). IL-1β and IL-18 are the major proinflammatory effectors produced in the NLRP3 signaling pathway and were elevated in SHR model rats (**Figure 2C** and **D**). To determine the gene expression of IL-1β and IL-18, RT‒qPCR was subsequently performed. Consistent with the increase in protein expression observed via western blotting, the mRNA levels of IL-1β and IL-18 were also pronounced in the SHR model group (**Figure 2E** and **F**). Taken together, these data indicate that NLRP3 signaling is activated in the brains of CSVD rats.

### MCC950 inhibits NLRP3 signaling in the brains of spontaneously hypertensive rats

**Figure 3:**
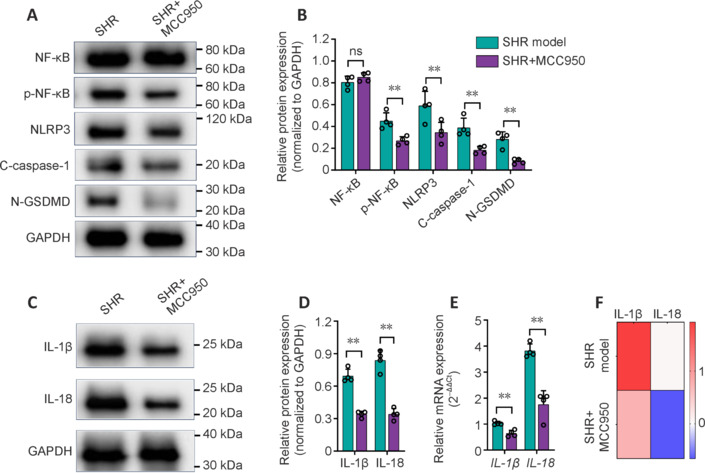
Effect of MCC950 on inactivating the NLRP3 inflammasome. (A, B) Representative immunoblots (A) and quantification (B) of brain expression of NF-κB, p-NF-κB, NLRP3, C-caspase-1 and N-GSDMD. (C, D) Representative immunoblots (C) and quantification (D) of IL-1β and IL-18 in the brain. (E) qRT-PCR analysis of the gene expression of *IL-1*β and *IL-18*. (F) qRT-PCR-based heatmap of IL-1β and IL-18 gene expression in the brains of SHRs and MCC950-treated SHRs. The data are shown as the mean ± SD. were compared via an unpaired *t* test. **P < 0.01, *n* = 4/group. All the experiments were repeated three times. C-caspase-1: Cleaved caspase-1; GAPDH: glyceraldehyde-3-phosphate dehydrogenase; IL-18: interleukin-18; IL-1β: interleukin-1 beta; NF-κB: nuclear factor kappa B; N-GSDMD: N-terminal gasdermin D; NLRP3: NOD-like receptor family, pyrin domain-containing protein 3; p-NF-κB: phosphorylated NF-κB; qRT-PCR: quantitative reverse transcription-polymerase chain reaction; SHR: spontaneously hypertensive rat; WKY: Wistar‒Kyoto rat.

To examine whether NLRP3 signaling in the brains of SHRs can be suppressed, MCC950 was then utilized to determine its impact on the expression of NLRP3 inflammasome elements and NF-κB. Using western blotting, we found that MCC950 substantially decreased the expression of NLRP3, cleaved caspase-1, N-GSDMD and P-NF-κB in the brains of the SHR-treated group compared with those of the SHR model group (*P* < 0.05; **Figure 3A** and **B**). Similarly, the protein expression levels of IL-1β and IL-18 were significantly lower in the SHR group than in the SHR model group (*P* < 0.05; **Figure 3C** and **D**). The results were further corroborated by qRT-PCR analysis, in which the mRNA levels of IL-1β and IL-18 were markedly lower in the MCC950-treated SHR group than in the SHR model group (*P* < 0.05; **Figure 3E** and **F**). These data confirmed the potent ability of MCC950 to suppress the NLRP3 signaling pathway in the brains of CSVD rats.

### MCC950 suppresses inflammation and glial activation in the brains of spontaneously hypertensive rats

**Figure 4:**
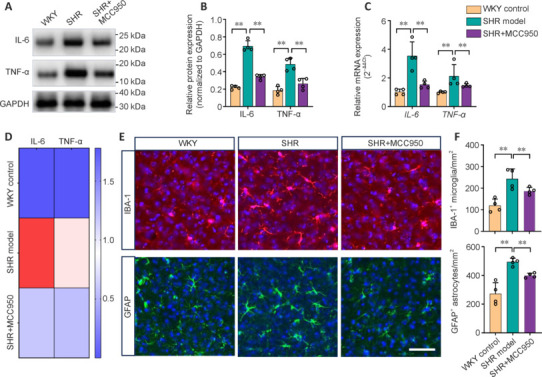
Effect of MCC950 on inhibiting inflammation in SHRs. (A) Representative immunoblots showing the expression of IL-6 and TNF-α in the brain. (B) Quantitative analysis of immunoblots showing the expression of IL-6 and TNF-α. (C) qRT-PCR analysis of the gene expression of *IL-6* and *TNF-α.* (D) qRT-PCR-based heatmap of *IL-6* and *TNF-α* gene expression. (E) Representative images of IBA-1 (red) and GFAP (green) staining in the deep cortical region. Scale bar: 40 μm. (F) Quantification of the histological assessment of the cell density of IBA-1^+^ microglia and GFAP^+^ astrocytes. The data are shown as the mean ± SD. **P < 0.01 (one-way analysis of variance followed by Tukey’s multiple comparisons test), *n* = 4/group. All the experiments were repeated three times. GAPDH: Glyceraldehyde-3-phosphate dehydrogenase; GFAP: glial fibrillary acidic protein; IBA-1: ionized calcium binding adaptor molecule 1; IL-6: interleukin 6; SHR: spontaneously hypertensive rat; TNF-α: tumor necrosis factor-alpha; WKY: Wistar‒Kyoto rat.

To further investigate the impact of MCC950 on the inflammatory response in the brain, the levels of pro-inflammatory factors downstream of NLRP3 signaling and resident immune cells were determined. Western blot analysis revealed a significant increase in the protein expression of IL-6 and TNF-α in the SHR model rats compared with the WKY control rats (*P* < 0.05; **Figure 4A** and **B**). MCC950 treatment diminished the excessive release of IL-6 and TNF-α (**Figure 4A** and **B**). In line with the western blot results, RT‒qPCR analysis revealed that the gene expression levels of IL-6 and TNF-α in the SHR model rats were markedly greater than those in the WKY control rats and were significantly lower in the MCC950-treated SHR rats (*P* < 0.05; **Figure 4C** and **D**). Additionally, immunohistochemical staining revealed that astrocytic and microglial activation (GFAP^+^ astrocytes and IBA-1^+^ microglia) in the brain (particularly in the corpus callosum and the deep cortical region adjacent to the corpus callosum) was markedly greater in the SHR model rats than in the WKY control rats. Extensive activation was dramatically inhibited by MCC950 treatment (**Figure 4E**). Quantitative analysis further revealed that the cell density of GFAP^+^ astrocytes and IBA-1^+^ microglia in the brains of the treated-SHR rats was markedly lower than that in the SHRs (*P* < 0.05; **Figure 4F**). These data indicate that the CSVD-affected brain is in a state of elevated inflammation, which further supports the activation of NLRP3 signaling in the CSVD brain. Moreover, the results demonstrated that MCC950 effectively mitigated neuroinflammation in CSVD SHRs by decreasing the levels of proinflammatory factors and glial activation.

### MCC950 inhibits autophagy in the brains of spontaneously hypertensive rats

**Figure 5:**
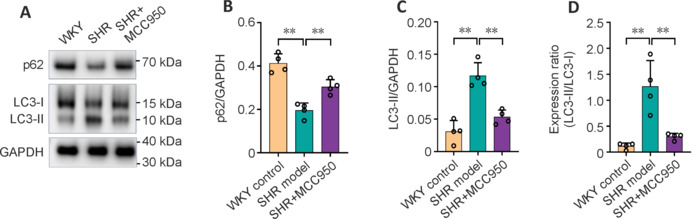
Impact of MCC950 on autophagy activity. (A) Representative immunoblots showing the expression of p62, LC3-I and LC3-II in the brain. Quantitative analysis of immunoblots showing the expression of p62 (B) and LC3-II (C). (D) Assessment of the conversion of LC3I to LC3II via the expression ratio of LC3-II/LC3-I. Data are shown as the mean ± SD. **P < 0.01 (one-way analysis of variance followed by Tukey’s multiple comparisons test), *n* = 4/group. All the experiments were repeated three times. GAPDH: Glyceraldehyde-3-phosphate dehydrogenase; LC3-I: microtubule-associated protein-I; LC3-II: microtubule-associated protein-II; SHR: spontaneously hypertensive rat; WKY: Wistar‒Kyoto rat.

p62 protein is an autophagic cargo receptor that recognizes ubiquitinated proteins and organelles, contributing to autophagosome formation. The accumulation of p62 indicates perturbed autophagy. Microtubule-associated protein light chain 3 (LC3)-II is generated by the conjugation of cytosolic LC3-I to phosphatidylethanolamine and is ultimately recruited to autophagosomal membranes. LC3-II is widely used as a marker for autophagosomes, and its accumulation indicates increased autophagy activity. We further investigated autophagy activity in the brains of SHRs by quantifying the levels of the autophagy markers p62 and LC3. As a result, we observed decreased p62 protein levels, increased LC3-II protein levels and protein expression ratios of LC3-II/LC3-I in the brains of SHR model rats compared with those in WKY control rats (*P* < 0.05; **Figure 5A–D**), indicating enhanced autophagic flux in the CSVD brain. Chronic MCC950 administration effectively inhibited autophagy, as demonstrated by the reduced accumulation of p62, increased accumulation of LC3-II and increased expression ratio of LC3-II/LC3-I in the treated SHR rats compared with the SHR model rats (*P* < 0.05; **Figure 5A–D**). The data revealed the potent effect of MCC950 on attenuating autophagy activity in the brains of CSVD rats.

### MCC950 ameliorates non-learning and memory deficits in spontaneously hypertensive rats

**Figure 6:**
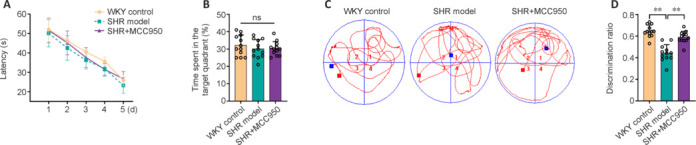
Effect of MCC950 on cognitive function in SHRs. (A) Analysis of escape latency to reach the hidden platform during the 5-day training. (B) The percentage of time spent in the target quadrant during the probe trial. (C) Representative exploratory paths in the probe test. The red square indicates the starting position, and the blue square indicates the ending position of the animal during the test. (D) Discrimination ability in the novel object recognition test. The data are shown as the mean ± SD. **P < 0.01 (repeated-measures analysis of variance for A and one-way analysis of variance followed by Tukey’s multiple comparisons test for B and D), *n* = 12/group. All the experiments were repeated three times. ns: not significant; SHR: spontaneously hypertensive rat; WKY: Wistar–Kyoto rat.

To evaluate the therapeutic effects of MCC950 as a pharmacologic intervention on cognitive performance, behavioral tests were conducted. The latency of the animals to find the platform in the MWM test was used to assess spatial learning. Consequently, no significant difference in the escape latency on the fifth day of the training test was observed among the three groups (**Figure 6A**). Spatial memory function was further assessed through a probe trial, where preference for the platform area was determined in the absence of the platform. Consistent with the escape latency, there was no significant difference among the three groups in the percentage of time spent in the target quadrant during this trial, which is a commonly used indicator of memory performance (**Figure 6B**). The MWM test results indicated that the SHRs did not exhibit learning or memory deficits. In contrast, NORT impaired nonspatial working memory in SHRs, as reflected by a lower discrimination ratio than in WKY control rats (*P* < 0.05; **Figure 6C**). In MCC950-treated rats, the discrimination ratio was significantly greater than that in SHR model rats (*P* < 0.05; **Figure 6D**), indicating that MCC950 improved the impairment of nonspatial recognition memory in CSVD rats.

### MCC950 alleviates neuropathology in spontaneously hypertensive rats

**Figure 7:**
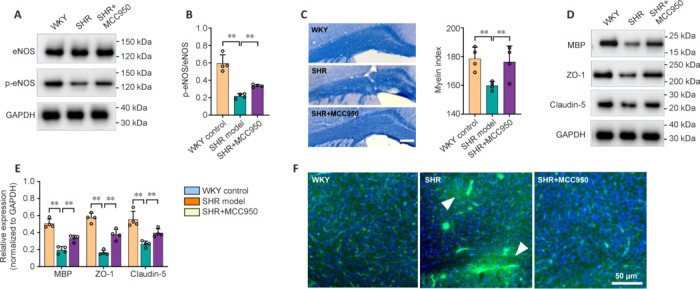
Effects of MCC950 on neuropathological alterations in SHRs. (A) Representative immunoblots showing the expression of eNOS and p-eNOS in the brain. (B) The ratio of p-eNOS/eNOS was determined via quantitative analysis of immunoblots. (C) Representative images of eriochrome cyanine staining of the corpus callosum showing myelin density, as reflected by the myelin index. Scale bar: 250 μm. (D, E) Representative immunoblots (D) and quantification (E) of the expression of MBP, ZO-1 and claudin-5 in the brain. (F) Representative images of albumin staining (green), with triangles indicating the location of albumin extravasation. The data are shown as the mean ± SD. **P < 0.01 (one-way analysis of variance followed by Tukey’s multiple comparisons test), *n* = 4/group. eNOS: Endothelial nitric oxide synthase; MBP: myelin basic protein; p-eNOS: phosphorylated eNOS; SHR: spontaneously hypertensive rat; WKY: Wistar-Kyoto rat; ZO-1: zonula occludens-1.

To elucidate the potential mechanisms underlying the therapeutic effects of MCC950, we further evaluated the typical pathophysiological events in the CSVD-affected brain, including the endothelium, white matter and BBB. Endothelial nitric oxide synthase (eNOS) is an important enzyme expressed in the endothelium that catalyzes the production of nitric oxide (NO). The activity of eNOS is widely used to represent endothelial function. The western blot results revealed that the ratio of P-eNOS (phospho-eNOS)/eNOS in the SHR model rats was significantly lower than that in the WKY control rats (*P* < 0.05; **Figure 7A** and **B**). The ratio was substantially greater in MCC950-treated SHRs than in SHRs (*P* < 0.05; **Figure 7A** and **B**). Eriochrome cyanine staining of the corpus callosum revealed myelin loss in the SHR model group compared with the WKY control group, as reflected by a slight but significant decrease in the myelin index (optical density) (*P* < 0.05; **Figure 7C**). MCC950 treatment reversed the decrease in the myelin index (**Figure 7C**). Similarly, the western blot results revealed a considerable decrease in the expression of MBP (myelin basic protein) in the SHR model rats compared with the WKY control rats (*P* < 0.05), which was markedly greater in the MCC950-treated SHR rats (*P* < 0.05; **Figure 7D** and **E**). To assess BBB integrity, the expression of tight junction proteins (ZO-1 and claudin-5) was quantified. Consequently, western blot analysis revealed markedly lower levels of ZO-1 and claudin-5 in the SHR model rats than in the WKY control rats (*P* < 0.05; **Figure 7D** and **E**). However, increased expression of these genes was observed after MCC950 intervention (**Figure 7D** and **E**). The disrupted BBB in the SHR model rats was further confirmed by histological observation of albumin extravasation in the brain, which was not detected following MCC950 treatment (**Figure 7F**). Taken together, these findings suggested that MCC950 alleviated neuropathology in CSVD rats by preserving the endothelial function and integrity of the white matter and the BBB.

## Discussion

We previously revealed the activation of NLRP3 signaling in the brain of SHR stroke-prone (SHRSP) rats as a CSVD model, suggesting that it is a major contributor to CSVD (Zhang et al., 2024). However, mechanistic investigations, which are essential for determining the role of NLRP3 in CSVD pathogenesis, are lacking. In addition, SHRSP rats develop malignant hypertension and typically die within several weeks due to their predisposition to cerebral bleeding, making them unsuitable for aging studies. Thus, in this study, we chose SHR rats as a model of CSVD and observed whether they presented a similar inflammatory profile as we reported in SHRSP rats. As a result, we detected high expression of NLRP3 and cleaved caspase-1 in the brains of SHRs, indicating the activation of NLRP3. Our results also revealed increased levels of the downstream pro-inflammatory effectors IL-1β, IL-18 and N-GSDMD, further supporting the activation of NLRP3 signaling in the CSVD-affected brain.

NF-κB, a ubiquitous transcription factor, is a key upstream molecule in the NLRP3 inflammatory pathway (Liu et al., 2023). The activation of NF-κB has been demonstrated to be the priming signal that triggers NLRP3 activation by upregulating the transcription of NLRP3. Our study revealed elevated expression of P-NF-κB, confirming the activation of NF-κB signaling in the CSVD brain. Furthermore, NF-κB activation can promote the production of pro-inflammatory cytokines (e.g., IL-18, IL-6, and TNF-α), which is in line with our observations in the present study (Naeem et al., 2024; Wei et al., 2024b). Glial cells, including astrocytes and microglia, are the primary brain-resident cells in the CNS and are the major players in neuroinflammation through the secretion of proinflammatory factors. Compelling studies have documented the central role of glial cells, especially reactive microglia (proinflammatory M1 phenotype), in various CNS diseases, including Alzheimer’s disease and Parkinson’s disease (Gao et al., 2023; Dai et al., 2024; Heuer et al., 2024). Our histological evidence revealed significant astrogliosis and activation of microglia in the brains of SHRs, which is consistent with previous studies reporting similar observations (Kaiser et al., 2014; Wei et al., 2024b). In addition, our study revealed high expression of M1-related microglial markers (e.g., IL-6 and TNF-α) (Salama et al., 2024; Wen et al., 2024), suggesting that M1-polarized microglia are predominant in the CSVD brain.

Many studies have demonstrated that MCC950 effectively attenuates NLRP3-driven inflammation, highlighting its promising therapeutic applications (Li et al., 2022a; Xu et al., 2024). In CNS diseases such as Alzheimer’s disease (Naeem et al., 2024), traumatic brain injury (Chakraborty et al., 2023) and multiple sclerosis (Hou et al., 2024), NLRP3-targeted therapy using MCC950 has shown great potential as an effective strategy to preserve structural integrity and improve neurological function. Our present study provides strong evidence that excessive inflammation in the CSVD brain is mediated by NLRP3 inflammasome signaling, indicating the potential of targeting NLRP3 as a therapeutic approach in CSVD. Therefore, we first examined whether MCC950 could inhibit NLRP3 activation and diminish the enhanced inflammatory response in the CSVD brain. As expected, our quantitative analysis revealed the potent ability of MCC950 to suppress extensive inflammatory activity, as exemplified by the reduced production of key molecules in the NLRP3 pathway (e.g., NLRP3 and cleaved caspase-1) and pro-inflammatory factors (IL-18 and IL-1β). Additionally, we also reported that MCC950 inhibited glial activation and prevented microglial polarization to the M1 phenotype, as evidenced by decreased expression of markers (IL-6 and TNF-α). These findings align with other studies, where MCC950 appears to modulate astrogliosis and polarization of microglia (Li et al., 2022b; Hou et al., 2024). The mechanisms underlying the influence of MCC950 on microglial polarization are not yet fully understood. However, increasing evidence has revealed the crucial role of the NF-κB pathway in M1 microglial activation (Zhang et al., 2013; Ganbold et al., 2020; Guo et al., 2022). Our study revealed that MCC950 treatment reduced the expression of P-NF-κB, a negative regulator of NF-κB signaling, suggesting a potential explanation for MCC950-mediated microglial polarization.

Dysregulation of autophagy, whether characterized by insufficient or excessive autophagy, results in cellular deficits and a decline in overall organismal function (Aman et al., 2021; Hernández-Cáceres et al., 2024; Nagayach and Wang, 2024). The dual effects (protective and detrimental) of autophagy have been well documented in CNS diseases, including ischemic stroke, Alzheimer’s disease and multiple sclerosis, which might be associated with the disease stage and severity or cell dependence (Minchev et al., 2022; Al-Kuraishy et al., 2024; Stanzione et al., 2024). Our quantitative analysis of autophagy markers revealed greater autophagy activity in SHRs than in age-matched WKY rats, indicating that autophagy was increased at the early stage of CSVD. Chronic MCC950 treatment substantially inhibited the activation of autophagy. These results are consistent with a recent report in which MCC950 reduced autophagy and improved cognitive function by inhibiting NLRP3-dependent neuroinflammation in Alzheimer’s disease (Naeem et al., 2024). Given that autophagy can suppress the NLRP3 inflammasome, we speculate that activated autophagy may represent a rescue mechanism with protective effects in response to stimuli, including molecules in the NLRP3 pathway. When NLRP3 signaling was significantly inhibited by MCC950, the activation of autophagy subsided afterwards. Such feedback is important for preventing the sustained activation of autophagy, as its overreaction may lead to cell stress or even cell death. Our hypothesis is in line with the view of Cadwell (2016), who proposed that this feedback loop is most obvious in the presence of inflammasome substrates to restore homeostasis. Taken together, our findings reveal the overall status of brain autophagy in early-stage CSVD. Further investigations are needed to further decipher the role of autophagy in CSVD progression at different stages and to identify specific cell types for the development of targeted autophagy-based therapies for CSVD.

We further investigated the therapeutic effect of blocking NLRP3 inflammatory signaling with MCC950 on cognitive function. The MWM test, a highly hippocampus-dependent behavioral test, was used in this study to evaluate spatial memory and learning ability. The MWM data revealed that the performance of the animals in each group was similar, indicating the intact functional integrity of the hippocampus in the SHRs at the age of 36 weeks. This result corroborated the findings of a previous study by Kaiser et al. (2014), where the same observation was reported using middle-aged SHRs (35 weeks of age) as a CSVD model. It is plausible that the cognitive decline resulting from CSVD particularly manifests as early executive function (rather than hippocampus-dependent memory impairment, such as in Alzheimer’s disease) but also reflects abnormal gait and depression. However, an earlier study revealed impaired structural integrity in 6-month-old SHRs, as evidenced by a reduction in gray matter volume in the hippocampal CA1 subfield and dentate gyrus (Sabbatini et al., 2002). One possible explanation is that the structural change in early-stage CSVD in middle-aged SHRs might not have reached the threshold beyond which hippocampus-related cognitive deficits occur. This hypothesis is supported by a recent clinical investigation in which the authors reported that hippocampal subfield atrophy was correlated with CSVD severity (Wong et al., 2021). Future studies, such as longitudinal assessments of hippocampal function and structure, are needed to further validate our speculation. In contrast to the MWM results, the discrimination ability of the NORT was lower in middle-aged SHRs than in age-matched WKY control rats. These data revealed the impairment of nonspatial recognition and memory in SHRs, which is consistent with other reports (Kaiser et al., 2014; Wen et al., 2024). Furthermore, we found that the discrimination ratio was markedly increased in the MCC950-treated group, suggesting that MCC950 could ameliorate cognitive deficits in SHRs.

To elucidate the potential mechanisms of cognitive improvement resulting from MMC950 treatment, we examined the neuropathological changes in the CSVD-affected brain, with a focus on endothelial function and the integrity of white matter and the BBB. Endothelial cells function as both structural and functional barriers between tissues and the blood. They modulate blood flow, regulate the transport of circulating components, and participate in inflammatory processes (Reiterer and Branco, 2020). In brain tissue, endothelial cells additionally serve as critical components of the BBB and neurovascular unit. Endothelial cell-secreted NO is an important signaling molecule that mediates vessel dilation in response to external stimuli, thereby regulating local cerebral blood flow (De Silva and Faraci, 2020). Endothelial dysfunction is considered an etiological contributor to CSVD (Quick et al., 2021). A reduction in NO release is a well-established indicator of endothelial dysfunction, resulting in pathological vasoconstriction, compromised cerebral blood flow, and ultimately, tissue ischemia (Theofilis et al., 2021). In this study, we investigated the activity of eNOS, an enzyme that converts L-arginine to NO, to evaluate the bioavailability of NO. Our quantitative analysis revealed decreased expression of P-eNOS in SHRs compared with that in age-matched WKY rats. However, this reduction was mitigated in MCC950-treated SHRs, highlighting the beneficial effect of MCC950 on maintaining endothelial function. This result is expected, as increasing evidence has revealed that proinflammatory mediators, including the NLRP3 inflammasome and TNF-α, are important driving forces of endothelial dysfunction (Zhang, 2008; Theofilis et al., 2021). Tight junctions, which are primarily composed of proteins such as ZO-1 and claudin-5, are vital structural and functional components of the BBB and are widely used as markers of BBB integrity (Sugiyama et al., 2023; Zheng et al., 2023). Our data revealed that the protein expression levels of ZO-1 and claudin-5 were decreased in SHRs, which was reversed in the MCC950-treated group. Histological evidence of albumin extravasation corroborated the compromised BBB in the brains of SHRs, and MCC950 treatment resulted in a marked reduction in this effect. These findings demonstrated the ability of MCC950 to preserve BBB integrity in the CSVD brain. Similarly, a recent investigation confirmed that MCC950 protected the integrity of the BBB with increased expression of tight junction proteins, resulting from a diminished inflammatory response (Cao et al., 2024). White matter degeneration is a hallmark feature of CSVD pathogenesis, leading to disrupted structural and functional connectivity in brain networks and a reduction in cortical thickness (Ter Telgte et al., 2018). Compelling evidence has revealed that WMH is associated with worse cognitive performance in patients with CSVD. Here, our histological assessment of the myelin sheath via eriochrome cyanine staining revealed white matter degeneration in SHRs compared with WKY rats. The microscopic staining results were similar for both groups, although our quantitative analysis revealed subtle yet significant differences. We hypothesize that at the age of 36 weeks, the animal model represents an early stage of CSVD, manifesting as mild histological alterations. To delve deeper into the phenomenon of myelin loss, we performed western blotting and quantified the expression of MBP, a major myelin sheath protein produced by mature oligodendrocytes. A significant decrease in the expression of MBP was subsequently revealed, corroborating the histological observation of myelin loss. More importantly, the changes were reversed by MCC950, indicating that MCC950 treatment could effectively restore the integrity of white matter.

This study has several limitations. First, brain tissue contains multiple cell types, such as neurons, glial cells and endothelial cells, and the composition of these cell types is altered during the course of disease (Itoh et al., 2018). In our study, the entire brain tissue was homogenized for quantifying protein and gene expression, which may confound mechanistic insights into the effects of specific cell types on disease progression. Future studies are needed to determine changes in protein and gene expression in a cell-specific and region-specific manner, which will improve the understanding of CSVD pathogenesis. Second, the WMW test was the only behavioral test used to assess spatial learning and memory in this study. Additional tests, such as the Barnes maze, would be valuable for further supporting conclusions regarding cognitive deficits. Our study also lacked a control group treated with the solvent alone. It is essential for elimination of any potential confounding effects of MCC950 treatment, even though PBS is not expected to have a therapeutic effect on CSVD. Future studies will address this limitation by incorporating a solvent-only control group to strengthen the reliability and robustness of our findings. Third, in this exploratory study, we set the endpoint at 36 weeks of age, representing early-onset CSVD, and investigated the therapeutic effects of MCC950 at a dose of 10 mg/kg body weight. While this dose demonstrated efficacy in our model, establishing a dose‒response curve to identify the optimal therapeutic window is important. Additionally, observation of the long-term therapeutic effects of MCC950 in halting CSVD progression is needed in future research. Notably, given the concern of MCC950-induced liver toxicity reported in a clinical trial (Li et al., 2022a), a comprehensive safety evaluation should be conducted over extended periods and across a range of doses to ensure the clinical relevance of MCC950. Fourth, future studies should consider employing *in vivo* imaging techniques such as clinically translational magnetic resonance imaging and positron emission tomography to provide spatiotemporal and quantitative assessments of neuroinflammation and brain ultrastructural and functional changes. These imaging modalities will enable direct comparisons between animal models and human CSVD patients, thereby improving the translational relevance of preclinical findings and facilitating the development of novel therapeutic strategies. Finally, while our study highlights the involvement of NLRP3 inflammasome activation in CSVD, further investigations into the potential of NLRP3 overactivation, particularly through genetic approaches (e.g., CRISPR/Cas9) or pharmacological manipulation to exacerbate neurocognitive deficits, are warranted. This would provide deeper insights into the role of NLRP3 in disease progression and may help to establish the broader therapeutic relevance of MCC950.

In summary, we reported that MCC950 effectively suppressed NLRP3 inflammasome-induced inflammation and autophagy in the brains of SHRs as a CSVD model. The treatment subsequently ameliorated cognitive impairment by improving endothelial function and the integrity of the BBB and white matter. These findings suggest the promise of targeting NLRP3 as a new therapeutic strategy in the context of CSVD.

## Data Availability

*All data relevant to the current study are included in the manuscript and available from the corresponding author upon reasonable request. We have also deposited the original WB images at Zenodo with the DOI (https://doi.org/10.5281/zenodo.14947666)*.
